# What strategies do desk-based workers choose to reduce sitting time and how well do they work? Findings from a cluster randomised controlled trial

**DOI:** 10.1186/s12966-018-0731-z

**Published:** 2018-10-12

**Authors:** Samantha K. Stephens, Elizabeth G. Eakin, Bronwyn K. Clark, Elisabeth A. H. Winkler, Neville Owen, Anthony D. LaMontagne, Marj Moodie, Sheleigh P. Lawler, David W. Dunstan, Genevieve N. Healy

**Affiliations:** 1 School of Public Health, The University of Queensland, Brisbane, Australia; 2 Baker Heart and Diabetes Institute, Melbourne, Australia; 3 Swinburne University of Technology, Melbourne, Australia; 4 Work, Health & Wellbeing Unit, Centre for Population Health Research, Deakin University, Geelong, Australia; 5 Deakin Health Economics, Centre for Population Health Research, Deakin University, Geelong, Australia; 6 School of Sport Science, Exercise & Health, University of Western Australia, Perth, Australia; 7 School of Exercise and Nutrition Sciences, Deakin University, Melbourne, Australia; 8 School of Public Health & Preventive Medicine, Monash University, Melbourne, Australia; 9 Mary MacKillop Institute for Health Research, Australian Catholic University, Melbourne, Australia; 10 School of Physiotherapy, Faculty of Health Sciences, Curtin University, Perth, Australia

**Keywords:** Workplace, Office workers, Sitting time, Sedentary, Physical activity, Intervention

## Abstract

**Background:**

Large amounts of sitting at work have been identified as an emerging occupational health risk, and findings from intervention trials have been reported. However, few such reports have examined participant-selected strategies and their relationships with behaviour change.

**Methods:**

The Stand Up Victoria cluster-randomised controlled trial was a workplace-delivered intervention comprising organisational, environmental and individual level behaviour change strategies aimed at reducing sitting time in desk-based workers. Sit-stand workstations were provided, and participants (*n* = 134; intervention group only) were guided by health coaches to identify strategies for the ‘Stand Up’, ‘Sit Less’, and ‘Move More’ intervention targets, including how long they would stand using the workstation. Three-month workplace sitting and activity changes (activPAL3-assessed total sitting, prolonged sitting (i.e., sitting ≥30 min continuously) and purposeful walking) were evaluated in relation to the number (regression analysis) and types of strategies (decision-tree analysis).

**Results:**

Over 80 different strategies were nominated by participants. Each additional strategy nominated for the ‘Stand Up’ intervention target (i.e. number of strategies) was associated with a reduction in prolonged sitting of 27.6 min/8-h workday (95% CI: -53.1, − 2.1, *p* = 0.034). Types of strategies were categorised into 13 distinct categories. Strategies that were task-based and phone-based were common across all three targets. The decision tree models did not select any specific strategy category as predicting changes in prolonged sitting (‘Stand Up’), however four strategy categories were identified as important for total sitting time (‘Sit Less’) and three strategy categories for purposeful walking (‘Moving More’). The uppermost nodes (foremost predictors) were nominating > 3 h/day of workstation standing (reducing total workplace sitting) and choosing a ‘Move More’ task-based strategy (purposeful walking).

**Conclusions:**

Workers chose a wide range of strategies, with both strategy choice and strategy quantity appearing relevant to behavioural improvement. Findings support a tailored and pragmatic approach to encourage a change in sitting and activity in the workplace. Evaluating participant-selected strategies in the context of a successful intervention serves to highlight options that may prove feasible and effective in other desk-based workplace environments.

**Trial registration:**

This trial was prospectively registered with the Australian New Zealand Clinical Trials register (ACTRN12611000742976) on 15 July 2011,

**Electronic supplementary material:**

The online version of this article (10.1186/s12966-018-0731-z) contains supplementary material, which is available to authorized users.

## Background

In light of consistent evidence showing that high levels of sitting (sedentary behaviour) are detrimentally associated with numerous health outcomes and premature mortality [1, 2], addressing this ubiquitous behaviour is now recognised as an important intervention target [3, 4]. A priority setting for addressing prolonged sitting (i.e., sitting for ≥30 min uninterrupted) is the desk-based workplace [5]. Desk-based workers typically sit for approximately 75% of their workday, with much of this sitting time accrued in prolonged unbroken bouts [6–8]. A growing number of trials have evaluated interventions to reduce sitting time in the office workplace [5]. In this context, interventions that utilise a multi-component approach (i.e. implementing behaviour change strategies across different levels of intervention such as individual and environmental change) to account for the multiple factors influencing sedentary behaviour [9], have typically been shown to be the most successful for reducing workplace sitting time in desk-based workers [5].

In addition to examining intervention efficacy, it is also important to ascertain *how* reductions in workplace sitting are achieved. Trials to reduce sitting time are still in their relative infancy, with intervention protocols often shaped by what is known to successfully increase levels of physical activity, i.e., self-monitoring of behaviour, changes to the physical environment, and use of prompts and cues [10–12]. However, sedentary behaviour has been identified as ubiquitous and habitual, differing from purposeful physical activity [13, 14], and as such, effective intervention approaches cannot be assumed to be the same [15]. In the context of workplace interventions to reduce sitting time, detailed consideration of strategies selected by participants and their relationship to sitting and activity changes is needed to inform future intervention development and workplace policies.

This study used data from the Stand Up Victoria workplace intervention trial, which achieved significant, substantial, and sustained reductions in workplace sitting time [16]. The activPAL3™ thigh-worn activity monitor (PAL Technologies Limited, Glasgow, UK) was used to measure sitting and activity outcomes. This device has been shown to be highly accurate and reliable for measuring sitting and sitting time accumulation patterns, as well as standing and stepping outcomes [17–19]. As previously reported [16], the intervention group achieved significant reductions in the primary outcome of workplace sitting time from baseline to three-month follow up: − 1.8 h/8 h at work (95% CI: -2.0, − 1.6). The sitting reduction was accompanied by a commensurate increase in workplace standing time, with minimal average change in time spent stepping (2.2 min/8 h at work) [16]. Much of the sitting change was from reducing the extent of sitting for 30 or more minutes continuously: − 1.5 h/8 h workday (95% CI: -1.7, − 1.3) [16].

The aim of this current study was to describe the strategies that Stand up Victoria intervention-arm participants identified to address the key intervention targets and examine their relationship to device-based behavioural outcomes reflecting the intervention targets.

## Methods

### Study design, participants and recruitment

Stand Up Victoria was a cluster-randomised controlled trial conducted in 2012–2014 in Victoria, Australia. The intervention complied with the CONSORT guidelines, and a populated CONSORT checklist is provided in Additional file 1. A populated TIDieR checklist for interventions is provided in Additional file 2. Previous reports have described the methods [20], intervention design [21], worksite characteristics [22], and primary activity outcomes [16]. In brief, 14 geographically separate worksites from a single organisation were recruited into the trial and randomised 50:50 to the intervention, or to act as control sites. The worksites from which participants were recruited varied in size from small (< 50 employees) to large (> 200 employees). The Alfred Health Human Ethics Committee (Melbourne, Australia) provided ethical approval for the Stand Up Victoria study, with written, informed consent provided at organisational, worksite and participant levels. In addition, ethical approval was granted by the University of Queensland, School of Public Health Research Ethics Committee (Brisbane, Australia) for these analyses. Only data from intervention participants with strategy choice recorded at baseline (*n* = 134 participants, 7 sites) were included in these analyses.

### Data collection and instrumentation

Study staff collected outcome and demographic data at baseline, three months (i.e. end of the health coaching and emails from managers), and 12 months. The strategy data used for the present analyses were collected by health coaches at baseline only. The focus of these analyses was on the effect of the strategies chosen at baseline on behaviour change at 3-month follow up. Detailed data on participant-selected strategy use was not collected at follow up. At each assessment, participants wore an activPAL3™ activity monitor continuously for seven consecutive days, and recorded their sleep/wake and work times in a diary. They also completed an online, self-administered questionnaire from which socio-demographic, work- and health-related data were collected, with demographic and work-related data collected at baseline only.

### Intervention

The primary aim of the trial was to reduce total workplace sitting time, with particular emphasis on reducing time spent in prolonged, unbroken bouts of sitting. The intervention comprised organisational, environmental, and individual-level components, which included consultation with managerial staff, a workplace information session, emails from worksite managers, installation of individually assigned sit-stand workstations, and individual health coaching [16, 20].

The three intervention targets — ‘Stand Up’, ‘Sit Less’, ‘Move More’ — were intended to reduce overall sitting time, particularly time spent sitting in prolonged, uninterrupted bouts of 30 min or more, and to increase time spent standing (accumulated across the workday) and moving (primarily through incidental activity) across the day during both work and non-work hours. The goal for the ‘Stand Up’ intervention target was to stand up at least every 30 min throughout the workday. The recommendation to interrupt sitting at least every 20 to 30 min was based on existing occupational health and safety recommendations [23] and findings from the experimental evidence emerging at the time of the trial [24, 25]. This messaging is consistent with the more recent American Diabetes Association (ADA) position statement on physical activity and sedentary behaviour [26]. Relatedly, the goal for the ‘Sit Less’ intervention target was to reduce total workplace sitting time by replacing some sitting with standing, primarily by using the workstation provided. The target for the amount of time spent standing during the work day was chosen by each participant, aiming for a gradual change towards approximately equal amounts of sitting and standing. The goal for the ‘Move More’ intervention target was to increase movement throughout the work day (without a quantified target), primarily through incidental activity [21].

### Health coaching

An initial face-to-face consultation session with a health coach trained in motivational interviewing was provided to all intervention participants following baseline assessment. Health coaches were guided by a script, which included provision of relevant information and prompts to assist in achieving the intervention targets. The session commenced with feedback for participants on their sitting and activity levels from the baseline assessment and the extent to which they were already meeting the intervention targets. Coaches then guided them to identify the specific, individual-level behaviour change strategies they could use to achieve these intervention targets, reflective of their personal preferences, job role and work environment. Participants were supported to identify and devise strategies which they considered to be potentially beneficial. A master list of possible strategies, including strategies identified by managerial staff as feasible for implementation in their workplace, were provided as examples where required. They were asked to identify two or three strategies for each of the ‘Stand Up’ and ‘Move More’ intervention targets. For example, *standing up when the phone rings* might be selected for the ‘Stand Up’ target and *using the stairs instead of the lifts* for the ‘Move More’ target. For the ‘Sit Less’ intervention target, participants were first asked to nominate an amount of time to stand using the sit-stand workstation provided (e.g. to stand for 60 min throughout the workday). They were then asked to identify two or three strategies to assist in achieving this goal, for example *reducing sitting time by standing for 30 min before or after lunch.* A written record of these strategies was attached to the participant’s sit-stand workstation for prompting and ongoing reference. They also received an individually tailored email with a summary of the session.

### Data preparation and coding: Strategies data

Data on participant-selected strategies to ‘Stand Up’, ‘Sit Less’, and ‘Move More’ were extracted from the initial health coach consultation feedback emails and entered into an Excel worksheet. Data were entered separately for each participant, with the strategies recorded for each intervention target. Data were initially copied verbatim as recorded in the feedback emails. This included the specific goal relating to the amount of time nominated to stand using the workstation (accumulated across the workday), and the strategies for each intervention target. The nominated accumulated time to stand using the workstation was collapsed into categories from 0 to 59, 60–119, 120–179 and ≥ 180 min per day. As the wording for each strategy was not standardised within the email feedback, individual strategies were initially collapsed into phrases identifying each unique strategy. For each intervention target, all unique strategies were classified into mutually exclusive categories based on the task (i.e., phone-based, emails), time (i.e., regular interruptions to sitting across the day), or prompt (i.e., environmental or colleague-based) involved. These categories were developed by several co-authors (SS, GH, EE and BC), and coding was completed by one coder (SS), with a random (10%) sample checked by a separate coder (BC) with initial 62.5% agreement. Within this random sample, consensus was achieved via discussion between co-authors (SS, BC, GH, EE) with 100% agreement. The number of strategies selected for each intervention target, and the total across all intervention targets, were counted.

### Data processing: activPAL3 activity monitor

The activPAL3 monitor data were processed in SAS 9.4 (SAS Institute Inc., Cary NC) as reported elsewhere [16]. The outcomes considered were total workplace sitting time, prolonged workplace sitting time (i.e., time spent sitting ≥30 min continuously), and purposeful walking time in the workplace (i.e., time stepping for ≥30 s continuously) [27]. These were selected as the markers best indicating that participants were ‘Standing Up’, ‘Sitting Less’, and ‘Moving More’ as per the key goals of these intervention targets, respectively. To reflect standing up regularly (i.e. interrupting prolonged periods of sitting), the prolonged sitting time outcome was chosen, rather than total standing time (which can occur in many ways). Likewise, purposeful walking was chosen for the ‘Move More’ target rather than total stepping, as the latter includes even minor stepping movements performed while participants are predominantly stationary (sometimes referred to as ‘stationary plus’ activity) [28]. Sleep, non-wear, non-work time, non-work days and invalid workdays were excluded. Outcomes used in this analysis were calculated for each participant across each day and averaged for valid workdays (monitor worn ≥80% of workplace time). To account for variations in wear time and work hours, variables were standardised to an eight-hour workday. For each time point, ≥ 1 valid workday was required for inclusion; and ≥ 1 valid workdays at both time points was required to assess change. Participants with valid data provided 1 to 6 valid workdays at baseline (mean = 4.2). Nearly all provided ≥3 valid workdays (99% at baseline, 97% at three months) and many provided ≥4 valid work days (88% at baseline and 83% at three months).

### Statistical analyses

**Fig. 1:**
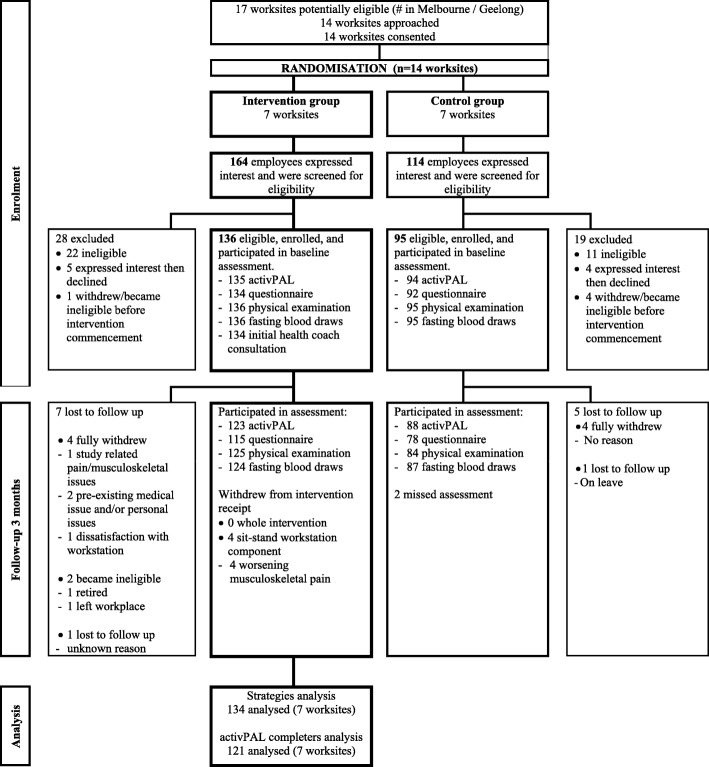
Flow diagram for enrolment, participation and analyses (baseline to 3-month follow up)

Statistical analyses were performed in SPSS Statistics Software, version 22 (SPSS, Inc., Chicago IL, USA) and STATA version 13 (STATACorp LP). Significance was set at *p* < 0.05 (two-tailed). Any interactions with *p* < 0.1 are reported. Missing data were excluded; analyses were of evaluable cases. Data on strategy use were available from 134 participants at baseline (Fig. 1). Complete activPAL3 data at baseline and 3-months were available from 121 participants. The frequency of selecting each strategy and the number of strategies selected were reported using descriptive statistics. Mixed models, adjusted for age and gender, tested the associations of number of strategies with changes in activity outcomes, correcting for cluster via a random intercept.

Decision tree models were used to identify the strategy categories and/or combination of strategy categories that discriminate between subgroups with the most and least successful behaviour change profiles [29]. To ensure effects of participant age, gender and worksite were accounted for, the outcome variables were adjusted for these characteristics using the residuals method, prior to their use in the decision tree models [30]. To provide at least 20 strategy users/non-users overall for the decision tree, only strategy categories nominated by at least 20 to 80% of participants were included. Strategies were all entered as binary, reflecting whether the participant nominated a strategy (or strategies) in the particular category or not. The sole exception was workstation targets, which were entered as the four categories described earlier, with the model determining the thresholds for the split within the decision tree. The classification and regression tree method was used [31]. Trees begin with all participants in the uppermost node, and the tree is ‘grown’, iteratively adding to the tree whichever predictor (here, strategy category) best separates the outcome across subgroups of participants (i.e. minimises within-node variance), up until a criterion for stopping (the tree reaches a pre-specified maximum depth, or nodes contain too few participants) is encountered [31]. The terminal nodes at the bottom of the tree indicate mutually exclusive subgroups of participants with a particular combination of exposures (here, strategy choices) that are, to some degree, predictive of the outcomes (e.g. the amount of sitting change that occurs). A commonly recommended approach was applied to elicit a large tree with small ‘leaves’, with the pruning method then applied to simplify the tree and avoid overfitting the model [31, 32]. Minimal improvement to the within-node variance (least-squared-deviation, 0.0001) was required to add predictors, and the default maximum depth was used (five layers), with minimum node sizes of five participants (both parent and child nodes) [31, 32]. The pruning then removed any predictors that did not sufficiently enhance the model’s prediction of the outcome (here, a criterion of one standard error (SE) of ‘risk’ was used) [29, 31, 32]. The decision trees are reported, along with the proportion of variance explained (PVE) by the model [29]. The PVE was calculated for each decision tree as 1 – (Within Node Variance/ Total Variance), or equivalently 1 – (risk estimate/ root node standard deviation (SD)^2^) [29]. A high PVE indicates the model is highly predictive of the outcome (within the sample).

## Results

### Participant characteristics

The characteristics of the Stand Up Victoria trial participants are described in detail elsewhere [16]. Briefly, the mean (± SD) age of intervention participants (*n* = 134) was 44.5 ± 9.0 years. Just under 80% (*n* = 106) of participants were employed at 1.0 full-time equivalent capacity, 66% (*n* = 88) were female, and 81% (*n* = 108) had a clerical, sales or service job role. At baseline, the average time spent per 8 h at work was 381 ± 49 min sitting, 68 ± 44 min standing and 31 ± 14 min stepping. On average, prolonged sitting comprised 215 ± 101 min of total sitting, and purposeful walking comprised 21 ± 13 min of stepping time.

### Types of strategies

In total, participants nominated 82 unique strategies to ‘Stand Up’ ‘Sit Less’ and/or ‘Move More’. The strategies were coded as belonging to 13 distinctive categories, as shown in Additional file 3. These strategy categories, as well as the strategies that were commonly identified (i.e., by at least 10% of participants) are described in Table 1. For the ‘Stand Up’ intervention target, strategies were nominated by participants in 10 of the 13 categories. The most common strategy category was prompts for phone-based tasks (e.g., stand up when the phone rings), with 55.9% of participants (*n* = 76) nominating at least one strategy within this category. Strategies pertaining to colleague-related prompts (50.0%), timer or clock prompts (47.1%), specific work tasks (41.9%), and ‘listening to your body’ (e.g. stand up when you feel discomfort) (31.6%), were also commonly nominated as a means of standing up regularly.

**Table 1:** Strategy categories (for each intervention target) nominated by Stand Up Victoria participants (*n* = 134)

Strategy category	Intervention targets, n (%)		
• Common strategies^a^	Stand Up	Sit Less	Move More
Phone-based strategies	76 (55.9)	21 (15.4)	1 (0.7)
• Stand up at the end of or after a phone call	34 (25.4)	–	–
• Stand up before making or taking a phone call	23 (17.2)	–	–
Work tasks	57 (41.9)	16 (11.8)	37 (27.2)
• Stand up after completing a task	29 (21.6)	–	–
• Walking to a colleague instead of emails	–	–	36 (26.9)
Listening to your body	43 (31.6)	8 (5.9)	65 (47.8)
• Stand up when you feel tired or uncomfortable	40 (29.9)	–	–
• Using glasses to drink water and filling up glass more regularly	–	–	35 (26.1)
• Drinking more water	–	–	29 (21.6)
Work environment	3 (2.2)	1 (0.7)	91 (67.9)
• Using the stairs more frequently	–	–	35 (26.1)
• Using a toilet further away from your desk	–	–	20 (14.9)
• Using a printer further away from your desk	–	–	18 (13.4)
Work breaks	1 (0.7)	24 (17.6)	76 (55.9)
• Standing for a defined block of time before or after lunch	–	17 (12.7)	–
• Walking during breaks	–	–	38 (28.4)
• Having lunch away from your desk	–	–	22 (16.4)
Colleague prompts	68 (50.0)	–	–
• Stand up when you see a colleague standing	54 (40.3)	–	–
Strategies promoting regular interruptions during the day	3 (2.2)	39 (28.7)	–
• Standing up at the start of the day	–	19 (14.2)	–
• Standing for regular blocks of time throughout the work day	–	14 (10.4)	–
Timer or clock prompts	64 (47.1)	–	–
• Stand at regular intervals using a timer, clock, stopwatch or alarm	59 (44.0)	–	–
Transport and commuting	–	–	23 (16.9)
• Parking your car further away	–	–	14 (10.4)
Work environment prompts	2 (1.5)	–	–
Self-monitoring	2 (1.5)	–	–
Maintenance of activity	–	–	4 (2.9)
Self-monitoring	–	–	1 (0.7)
Social support prompts	–	–	1 (0.7)

Table reports number (n) and percentage (%) of intervention participants who nominated at least one strategy of each of the types above

^a^Strategy nominated by at least 10% of intervention participants

For the ‘Sit Less’ intervention target, strategies were nominated across six categories (Table 1). The most common strategy categories were regular interruptions to sitting time across the day (nominated by 28.7% of participants), work breaks (17.6%), and phone-based tasks (15.4%). All participants (100%) set a goal in relation to standing at their workstations: 27.9% (*n* = 38) for ≥3 h; 45.5% (*n* = 61) nominated to stand for 2–3 h; 19.9% (*n* = 27) for 1–2 h; and 6.7% (*n* = 8) < 1 h.

For the ‘Move More’ target, strategies were nominated across nine categories (Table 1). Strategies pertaining to the work environment (e.g., stair use, using a centralised bin or printer) were the most common, with 67.9% of participants (*n* = 91) nominating at least one strategy in this category. Other common strategy categories for this target were: work breaks (55.9%); listening to your body (47.8%); task-based prompts (27.2%); and, transport and commuting (16.9%).

### Number of strategies

Participants nominated 0–4 unique strategies per intervention target and 2–8 strategies in total across all intervention targets, with a mean (± SD) of 5.8 ± 1.5 strategies. The only statistically significant association between number of strategies and behaviour change was for the ‘Stand Up’ intervention target. Here, each additional ‘Stand Up’ strategy nominated was associated with a reduction in prolonged sitting of 27.6 min/8-h workday (95% CI: -53.1, − 2.1, *p* = 0.034) as shown in Table 2. Other associations were smaller, and not statistically significant, but were estimated with wide confidence intervals.

**Table 2:** Associations of number of types of nominated strategies with 3-month changes in workplace activity (*n* = 121)

Change in workplace activity (baseline to 3 months)	Total (2–8)		‘Stand Up’ (1–4)		‘Sit Less’ (0–3)		‘Move more’ (1–4)	
	Coefficient (95% CI)	*p*	Coefficient (95% CI)	*p*	Coefficient (95% CI)	*p*	Coefficient (95% CI)	*p*
Total workplace sitting time, *min/8-h workday*	1.5 (−13.0, 16.0)	0.841	−0.2 (−28.0, 27.7)	0.991	−1.8 (−26.3, 22.7)	0.885	5.0 (− 17.1, 27.2)	0.655
Prolonged sitting bouts ≥ 30 min, *min/8-h workday*	−11.8 (− 25.2, 1.6)	0.083	**−27.6 (−53.1, − 2.1)**	**0.034**	−10.7 (−33.5, 12.2)	0.360	1.4 (− 22.2, 19.3)	0.890
Continuous stepping ≥ 30 s, *min/8-h workday*	0.4 (−0.8, 1.5)	0.540	0.3 (−2.0, 2.5)	0.818	−1.9 (− 2.5, 2.2)	0.871	0.9 (− 1.0, 2.8)	0.334

Table reports regression coefficient adjusted for age and gender, and 95% confidence intervals (CI) obtained from mixed models, with a random intercept for cluster (worksite). Associations with *p* < 0.05 are shown in boldface

### Types of strategies that predicted successful behaviour changes

The decision tree model for prolonged sitting time did not retain any of the strategy categories, as no strategy category, in particular, predicted a successful change in behaviour (up to the pruning criteria). The PVE was 0% as the model contained no predictors to explain variation.

**Fig. 2:**
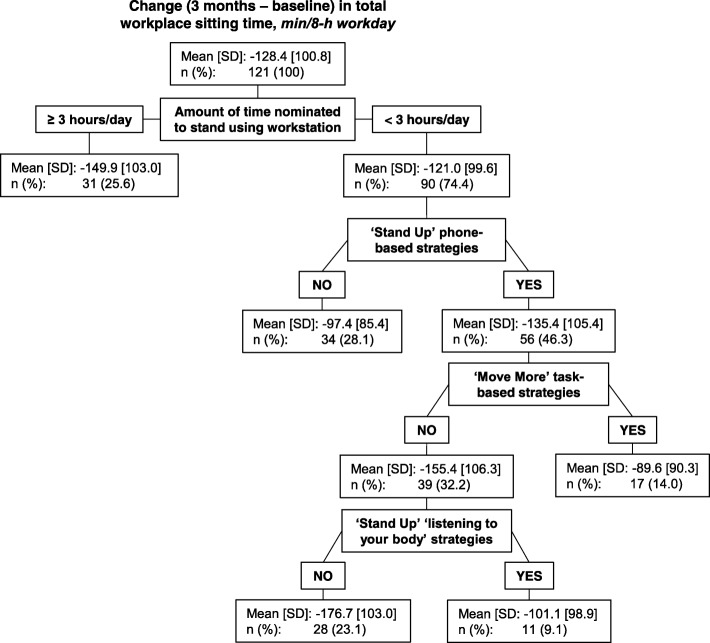
Decision tree depicting subgroups with differing levels of change in total workplace sitting time

Figure 2 shows the decision tree for workplace sitting time (achieving the ‘Sit Less’ target). The model explained only a small proportion of variance in total sitting at work (12.7%). Nominating to stand using the workstation for ≥3 h as opposed to < 3 h was the uppermost node. The other strategy categories that also contributed to predicting greater sitting time reductions were using phone-based strategies to ‘Stand Up’, not nominating task-based strategies to ‘Move More’, and not nominating ‘Stand Up’ listening to your body strategies. All of the subgroups experienced some reduction in sitting time, however mean reductions over two hours were evident in those who had a high workstation target; or, had a lower workstation target, and nominated ‘Stand Up’ phone-based strategies, while not nominating both task-based strategies to ‘Move More’ and listening to your body strategies to ‘Stand Up’.

**Fig. 3:**
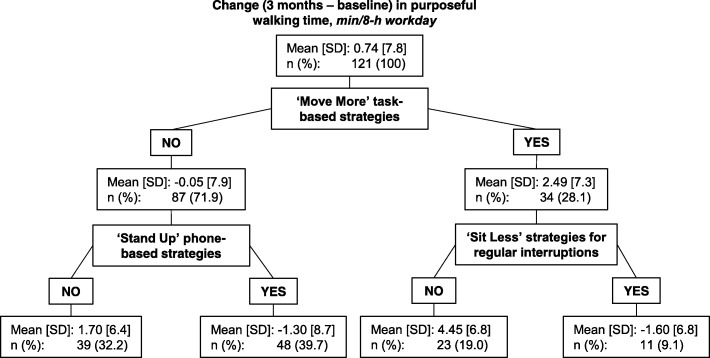
Decision tree depicting subgroups with differing levels of change in purposeful walking time

Figure 3 shows the decision tree for purposeful walking time (achieving the ‘Move More’ target). Only a small proportion of variance was explained by the model (9.1%). The uppermost node was nominating task-based strategies to ‘Move More.’ The other predictors of an increase in purposeful walking time were not nominating strategies pertaining to regular interruptions to ‘Sit Less’ and not nominating phone-based strategies to ‘Stand Up’. Of the four subgroups identified by the decision tree, the largest average improvement (4.5 ± 6.8 min) was in those who nominated at least one ‘Move More’ task-based strategy, but did not nominate strategies to promote regular interruptions throughout the workday. Very small average changes (from − 1.6 min to + 1.7 min) were seen in the remaining subgroups.

## Discussion

The Stand Up Victoria intervention achieved an average reduction in total workplace sitting time of more than one and a half hours and prolonged sitting time of more than one hour at the 3-month follow up in the intervention group compared to controls [16]. Here, we have examined the strategies that participants identified (with guidance from a health coach) to address the key intervention targets of ‘Stand Up’, ‘Sit Less’ and ‘Move More’; and their relationship to corresponding objectively measured behavioural outcomes. Participants nominated over 80 strategies, and all participants chose an amount of time to stand at a sit-stand work station. The number of strategies nominated was associated with changes in prolonged sitting time, with greater reductions seen in those who nominated more strategies. The types of strategies chosen (strategy categories), in particular, phone-based and task-based strategies to ‘Stand Up’ and ‘Move More’, appeared to be relevant to changes in total workplace sitting and purposeful walking. Such understanding may assist in identifying approaches for future desk-based workplace interventions that may be acceptable and feasible.

Workers, most of whom had a clerical, sales or service job role, collectively nominated a wide array of strategies to ‘Stand Up’, ‘Sit Less’ and ‘Move More’. Most participants indicated they planned to stand for at least two hours, accumulated across the workday, using the workstation. Given the large average reduction in workplace sitting time, these findings demonstrate that participants perceive such substantial changes to be acceptable, and that they are largely achievable. Task-based and phone-based types of strategies as well as strategies pertaining to work breaks and listening to your body were common across each of the intervention targets. This is in line with previous research, with strategies such as these reported to be regularly used [33, 34] and potentially feasible to implement [35, 36] in interventions to reduce workplace sitting time. The popularity of nominating phone-based prompts suggests this was highly appropriate for the tasks undertaken in the workers job roles. This supports the importance of using strategies that are incorporated into common job tasks, which workers will have high exposure to across the day. It demonstrates the need for a tailored and worker-specific approach to proactive strategy development to enable individual workers and workplaces to incorporate strategies that are suitable in the context of the particular jobs and work environment they are being implemented in. Further, workplace policies will need to be adaptive to the high number and variation in strategies that can be used.

For the ‘Stand Up’ target, nominating a higher number of strategies was associated with significantly greater reductions in prolonged sitting of over 25 min per additional strategy nominated. For this key outcome, none of the most commonly nominated strategy categories were retained in the decision tree model, suggesting that no single strategy category or combination thereof were strong predictors of successful behaviour change. This may indicate that in order to achieve a reduction in prolonged bouts of sitting time (and therefore increasing standing across the work day), nominating multiple various strategies (i.e., a higher number of strategies) may be more beneficial than nominating a particular type of strategy, and that varying combinations may be required to suit individual jobs and workers. Identifying multiple opportunities to stand throughout the day (i.e., break up prolonged sitting) for example, prompts to stand when the phone rings or at the completion of a task, are likely to increase the chances of these opportunities occurring during a usual workday.

The number of strategies used was not significantly associated with changes in total workplace sitting time and purposeful walking at work. In contrast, several strategy categories were retained in the decision tree models as discriminating between groups of participants with better and worse change profiles for these outcomes. The types of strategies that participants nominated appeared to have some relevance to their degree of attainment of the ‘Sit Less’ and ‘Move More’ targets. The strategy categories that featured prominently in the models were those that were consistent with what the workers were likely to do regularly within their job role (i.e., phone and task-based strategies primarily for the ‘Stand Up’ and ‘Move More’ targets). It appears that selecting longer workstation standing time may be particularly important to reduce total sitting time at work (even though all participants were provided with a sit-stand workstation). The benefit may have to do with the process of goal setting for this workstation use strategy, the motivation of the participant to stand more, or the opportunity to use the workstation as part of daily work tasks. Similarly, selecting task-based strategies to ‘Move More’ appeared important to increase purposeful walking. While there was a small degree of ‘trade-off’ between strategies enhancing ‘Sit Less’ while detracting from ‘Move More’ or vice versa, it is important to note that differences in purposeful walking were very small, with all sub-groups of participants (i.e. in each of the terminal nodes of the decision tree in Fig. 3) differing by less than 10 min per day. For changes in total workplace sitting time, however, the sub-groups of participants (in Fig. 2), showed sizeable average changes in sitting time of around an hour and a half per day.

This is one of the first studies to comprehensively report on the types of strategies that participants identified to reduce workplace sitting and the only study to examine the relationship between these strategies and device-based activity change during the intervention. It is important to note, that the findings from this study are not able to be generalised to outcomes which may occur in work environments that do not include the use of sit-stand workstations, as this was a key component of the Stand Up Victoria intervention. Both the choice of strategy, and effectiveness of the chosen strategies, may be affected by the availability of a workstation or other prompts to change sitting behaviour. For example, one study, which examined the uptake of strategies in a non-workstation based intervention identified the use of both similar (e.g. walking to a lavatory or shared space further away from the desk, and the use of computer-assisted prompts) and different (e.g. standing and walking meetings) strategies to those identified in this study [33]. As such, this again supports a tailored approach to strategy selection, and highlights the need for further evaluation of the use of strategies in both workstation and non-workstation interventions.

As this study involves secondary analyses, this intervention was not powered a priori for these analyses and, based on the wide confidence intervals, may have missed sizeable associations between behaviour change and the number of strategies participants nominated. The low PVE for the decision tree models indicated strategy categories had limited ability to predict outcomes within the Stand Up Victoria participant sample; predictive ability in other populations would therefore be limited. Further, all participants were recruited from a single organisation, also limiting generalisability. Importantly, as this study was exploring the types of strategies nominated by participants, due to the limitations in data available, we were not able to determine the extent to which these strategies were actually used at follow up. In the future, participant identified strategies should be captured both prospectively and retrospectively to inform the development, use and uptake of strategies in achieving a change in workplace sitting time.

## Conclusions

This study examined the predictive effects of tailored strategy development on achieving behaviour change, finding that, whether specifically implemented across the study period or not, both strategy choice and strategy quantity appeared relevant to behavioural improvement. This has important implications for future strategy development and implementation, where the focus may be substantially on coaching and facilitating which targets and strategies are able to motivate workers to achieve a change in workplace sitting behaviour. Evaluating the strategies that desk-based workers selected to implement in a successful intervention serves to highlight options that may prove feasible and effective within other desk-based workplace environments.

These findings suggest that selecting strategies appropriate to the workplace environment and work tasks, and to the outcomes targeted in the intervention were relevant to achieving intervention change. This further supports the need for a tailored and pragmatic approach to encourage a change in sitting and activity in the workplace. In these workers, phone-based and task-based strategies, as well as strategies relating to work breaks and ‘listening to your body’ were commonly chosen. A key next step in future research is to examine which strategies participants used, through for example, self-report questionnaire data in combination with examination of detailed data from activity monitors on the nature and timing of changes.

## Take home messages

- Participant’s should be able to choose what strategies work for them in the context of their work environment and work tasks
- Identifying strategies that are individualised, pragmatic, and context-specific is important for achieving a change in sitting behaviour
- Enabling multiple opportunities to stand across the workday, accounting for the variability and number of strategies that may be used, is likely to enhance implementation of strategies into a participant’s usual work day

## Additional files

Additional file 1: CONSORT 2010 checklist of information to include when reporting a cluster randomised trial. Populated CONSORT Extension for Cluster-Randomised Trials 2012 Checklist. (DOCX 29 kb)

Additional file 2: The TIDieR (Template for Intervention Description and Replication) Checklist. Populated TIDieR Checklist. (DOCX 29 kb)

Additional file 3: **Table S1.** Strategies by category for each of the intervention targets. This table reports all unique strategies nominated by intervention participants (*n* = 134) for each intervention target, within each strategy category. (DOCX 20 kb)
