# Supplementary material for: What strategies do desk-based workers choose to reduce sitting time and how well do they work? Findings from a cluster randomised controlled trial
Source: Int J Behav Nutr Phys Act. 2018 Oct 12;15:98. doi: 10.1186/s12966-018-0731-z (PMC6186123; doi:10.1186/s12966-018-0731-z)
Supplement: Supplementary file 3 — Table S1. Strategies by category for each of the intervention targets. This table reports all unique strategies nominated by intervention participants (n = 134) for each intervention target, within each strategy category. (DOCX 20 kb) [file 12966_2018_731_MOESM3_ESM.docx]

**Supplemental Table 1.** Strategies by category for each of the intervention targets.

| **Category** | **‘Stand Up’ Intervention Target** | **‘Sit Less’ Intervention Target** | **‘Move More’ Intervention Target** |
| --- | --- | --- | --- |
| Phone-based | - Stand up before making or taking a phone call - Stand up during a phone call - Stand up after a phone call - Stand up when on hold on the phone - Stand up in between phone calls - Stand up when the phone rings (not necessarily yours) - Monitor frequency of phone calls and stand up at nominated times (e.g. after every 3^rd^ phone call) | - Stand up during a phone call - Stand up after a phone call - Monitor frequency of phone calls and stand up at nominated times (e.g. stand after every 3^rd^ phone call) - Alternating sitting and standing at nominated times relating to phone calls | - Going for a short walk after each call |
| Task-based | - Stand up when you commence a task - Stand up during a task - Stand up after completing a task - Monitor frequency of tasks completed (e.g. stand up after every 3^rd^ task) - Stand up during meetings - Stand up when checking or sending emails - Stand up when you get a message (instant messaging program) | - Standing during meetings - Stand up when checking or sending emails - Stand up during a task - Alternating standing and sitting for nominated work tasks | - Standing during meetings - Walking to a colleague rather than using emails |
| Work environment | - Stand up because you have a workstation that allows you to - Leave your desk in the standing position when you go for breaks | - Leaving your desk in the standing position when you go to breaks | - Removing use of bin in workspace - Purchasing some resistance bands - Take the longer route around the office - Take each letter to the letterbox - Taking mail around to the mail room - Using a printer (picking up printing) further away from your desk - Using a bin further away from your desk (e.g. centralised bin) - Picking up printing more often - Use a refrigerator further away from workspace - Disposing of waste more frequently - Using the stairs more frequently - Using the stairs at specific times of the day (e.g. start or end of the day) - Nominating levels of the building to use the stairs between floors - Using a toilet further away from your desk - Walking around at your desk |
| Listening to your body | - Stand up when you feel tired and/or uncomfortable - Stand up when you need to stretch - Stand up when drinking water | - Changing position (e.g. sitting or standing) when you feel discomfort or tired - Standing at the start of the day until you feel tired - Standing after lunch until you feel tired - Standing after afternoon tea break until you feel tired | - Using glasses to drink water and filling up glass more regularly - Drinking more water - Taking bathroom breaks (or more bathroom breaks) throughout the day |
| Work breaks | - Stand up at morning tea | - Standing for a defined block of time after morning tea - Standing for a defined block of time before or after lunch - Standing for a defined block of time after afternoon tea - Standing times around breaks | - Standing during breaks - Walking during breaks - Nominating defined block of time to walk during breaks - Active breaks (e.g. gym) - Having lunch away from your desk (e.g. outside) |
| Self-monitoring | - Using a checklist/timetable - Seeing your tracker |  | - Wearing a pedometer to keep track of your steps throughout the day |
| Work environment prompts | - Stand up at news breaks on the radio (every 30 minutes) - Stand up at ad break on the radio |  |  |
| Strategies promoting regular interruptions during the day | - Stand up every 30 minutes - Stand up at regular intervals (e.g. every half an hour) - Take breaks at well-spaced intervals throughout the day | - Standing up for a defined block of time at the start of the day - Standing up for defined blocks of time in the afternoon - Standing up for regular blocks of time during the day - Alternating standing and sitting regularly throughout the day |  |
| Colleague prompts | - Stand up when you see a colleague standing - Stand up when you visit colleagues at their desks - Stand up when someone enters your workspace - Stand up when somebody greets you - Stand up when a colleague picks up copies from the photocopier - Stand up when a colleague notifies you that they are going for a break |  |  |
| Use of timer or clock prompts | - Stand at regular intervals using a timer, clock, stopwatch or alarm (e.g. every 30 minutes) - Stand up when Pulse Machine hits red |  |  |
| Transport and commuting |  |  | - Walking before, to or from work - Parking your car further away - Nominating to vary transport to or from work to include more walking (e.g. different train station) - Riding to or from work |
| Maintenance of activity |  |  | - Continuing to be active throughout working day |
| Social support prompts |  |  | - Having a walking buddy for breaks - Social walking group |
